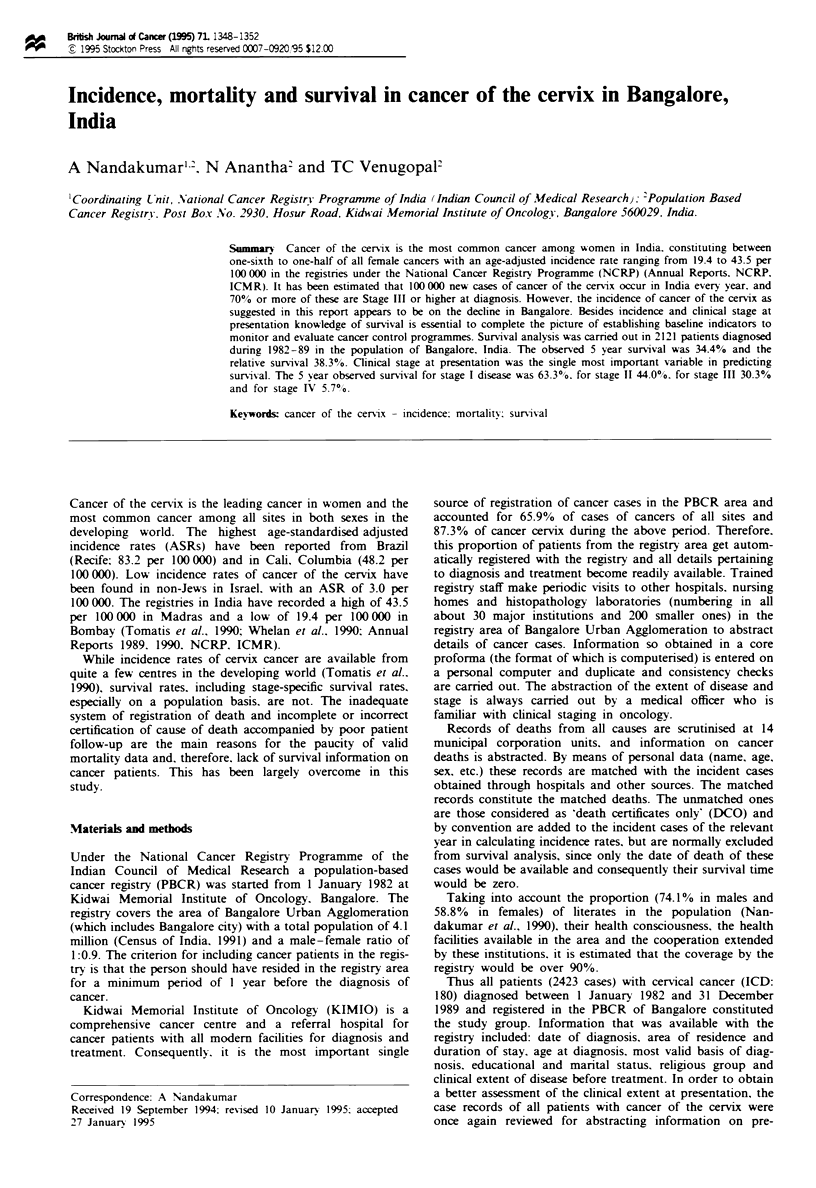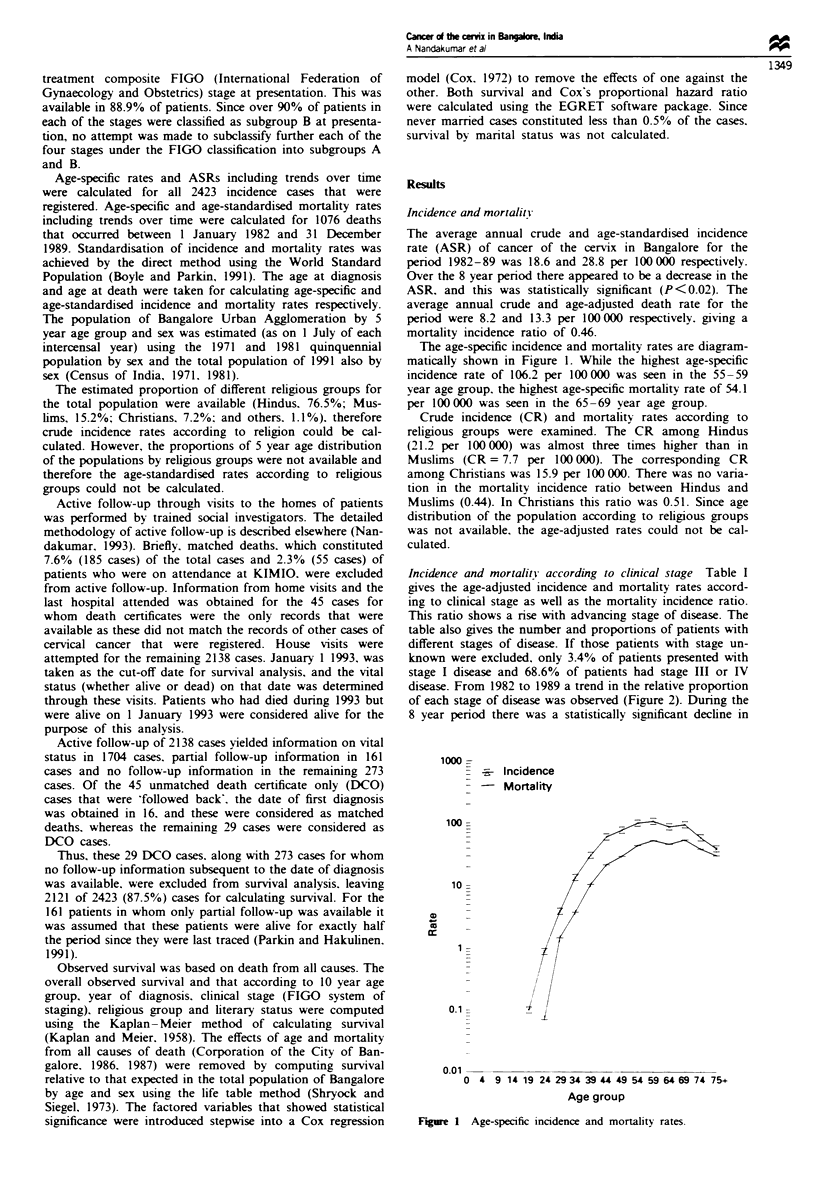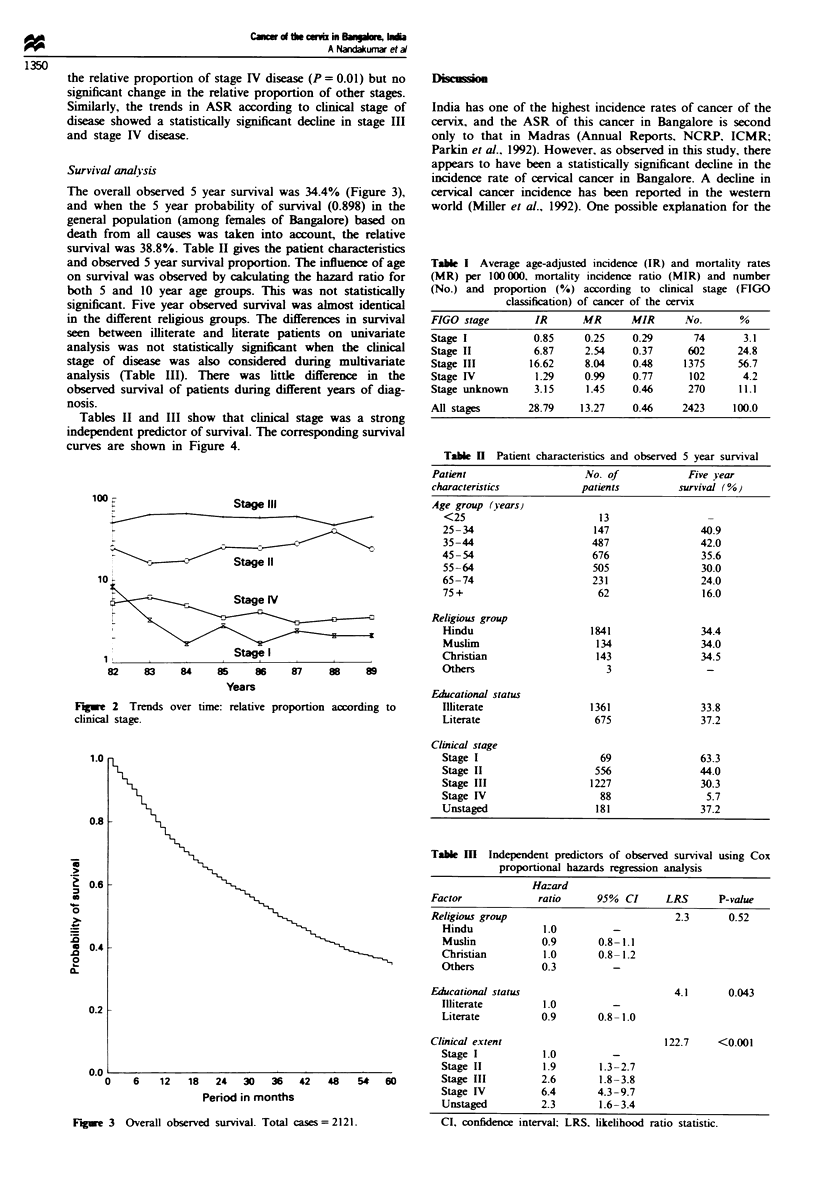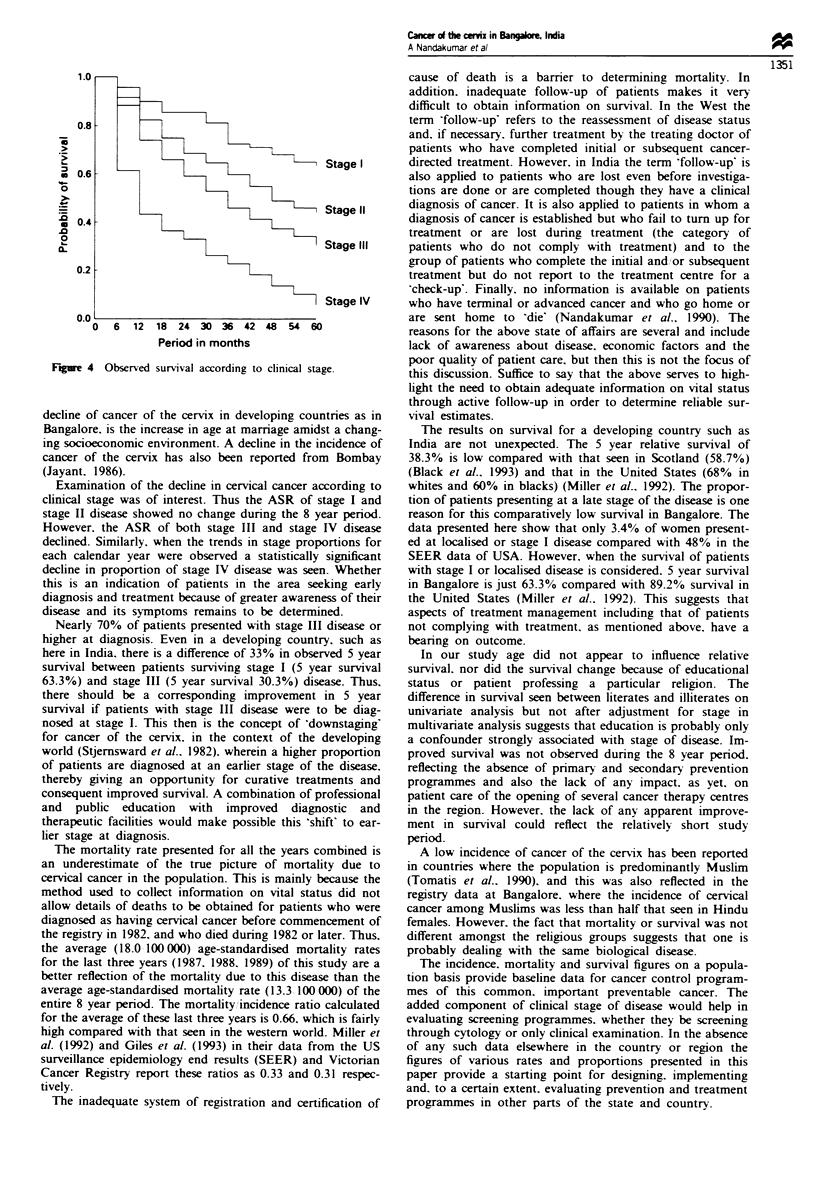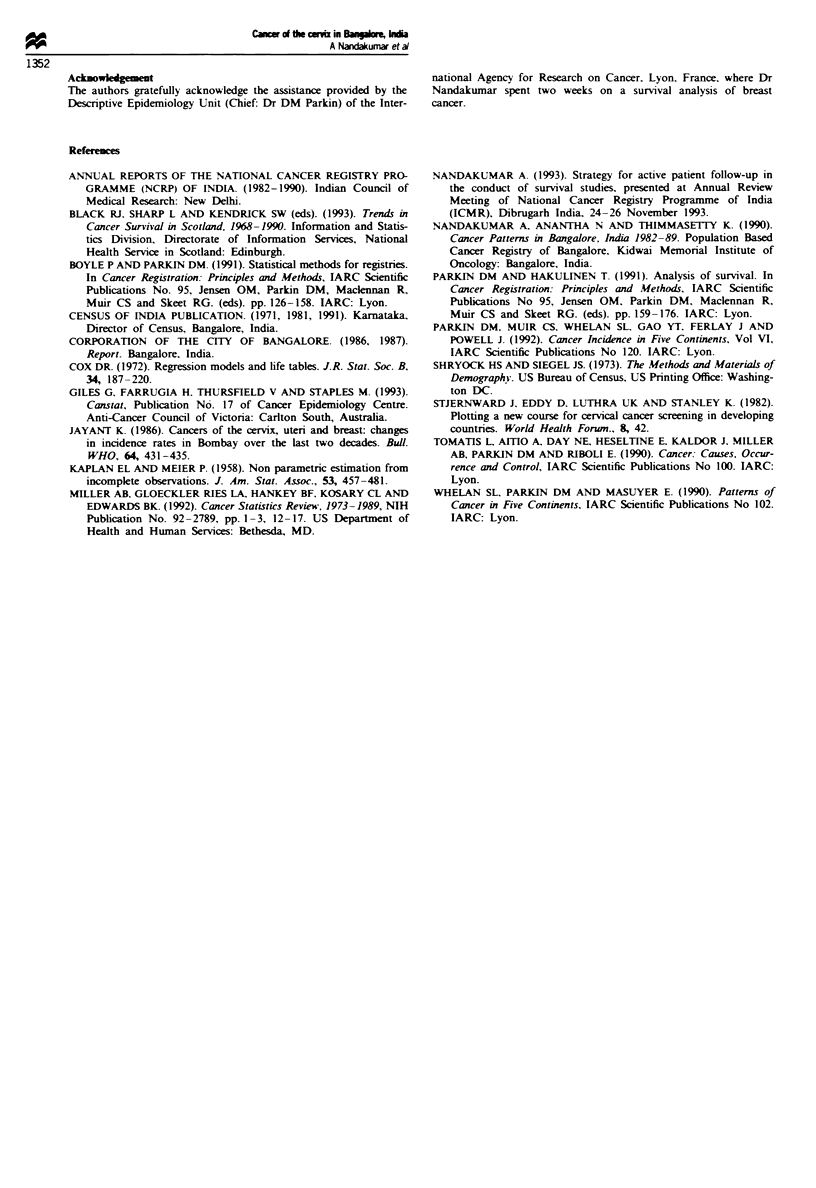# Incidence, mortality and survival in cancer of the cervix in Bangalore, India.

**DOI:** 10.1038/bjc.1995.262

**Published:** 1995-06

**Authors:** A. Nandakumar, N. Anantha, T. C. Venugopal

**Affiliations:** Coordinating Unit, National Cancer Registry Programme of India (Indian Council of Medical Research), Kidwai Memorial Institute of Oncology, Bangalore.

## Abstract

Cancer of the cervix is the most common cancer among women in India, constituting between one-sixth to one-half of all female cancers with an age-adjusted incidence rate ranging from 19.4 to 43.5 per 100,000 in the registries under the National Cancer Registry Programme (NCRP) (Annual Reports, NCRP, ICMR). It has been estimated that 100,000 new cases of cancer of the cervix occur in India every year, and 70% or more of these are Stage III or higher at diagnosis. However, the incidence of cancer of the cervix as suggested in this report appears to be on the decline in Bangalore. Besides incidence and clinical stage at presentation knowledge of survival is essential to complete the picture of establishing baseline indicators to monitor and evaluate cancer control programmes. Survival analysis was carried out in 2121 patients diagnosed during 1982-89 in the population of Bangalore, India. The observed 5 year survival was 34.4% and the relative survival 38.3%. Clinical stage at presentation was the single most important variable in predicting survival. The 5 year observed survival for stage I disease was 63.3%, for stage II 44.0%, for stage III 30.3% and for stage IV 5.7%.


British Journal of Cancer (1995) 71, 1348-1352

© 1995 Stockton Press All rights reserved 0007-0920/95 $12.00

### Incidence, mortality and survival in cancer of the cervix in Bangalore, India

A Nandakumar[1,2], N Anantha[2] and TC Venugopal[2]

[1]*Coordinating Unit, National Cancer Registry Programme of India (Indian Council of Medical Research); [2]Population Based Cancer Registry, Post Box No. 2930, Hosur Road, Kidwai Memorial Institute of Oncology, Bangalore 560029, India.*

**Summary** Cancer of the cervix is the most common cancer among women in India, constituting between one-sixth to one-half of all female cancers with an age-adjusted incidence rate ranging from 19.4 to 43.5 per 100 000 in the registries under the National Cancer Registry Programme (NCRP) (Annual Reports, NCRP, ICMR). It has been estimated that 100 000 new cases of cancer of the cervix occur in India every year, and 70% or more of these are Stage III or higher at diagnosis. However, the incidence of cancer of the cervix as suggested in this report appears to be on the decline in Bangalore. Besides incidence and clinical stage at presentation knowledge of survival is essential to complete the picture of establishing baseline indicators to monitor and evaluate cancer control programmes. Survival analysis was carried out in 2121 patients diagnosed during 1982-89 in the population of Bangalore, India. The observed 5 year survival was 34.4% and the relative survival 38.3%. Clinical stage at presentation was the single most important variable in predicting survival. The 5 year observed survival for stage I disease was 63.3%, for stage II 44.0%, for stage III 30.3% and for stage IV 5.7%.

**Keywords:** cancer of the cervix - incidence; mortality; survival

Cancer of the cervix is the leading cancer in women and the most common cancer among all sites in both sexes in the developing world. The highest age-standardised adjusted incidence rates (ASRs) have been reported from Brazil (Recife; 83.2 per 100 000) and in Cali, Columbia (48.2 per 100 000). Low incidence rates of cancer of the cervix have been found in non-Jews in Israel, with an ASR of 3.0 per 100 000. The registries in India have recorded a high of 43.5 per 100 000 in Madras and a low of 19.4 per 100 000 in Bombay (Tomatis *et al.*, 1990; Whelan *et al.*, 1990; Annual Reports 1989, 1990, NCRP, ICMR).

While incidence rates of cervix cancer are available from quite a few centres in the developing world (Tomatis *et al.*, 1990), survival rates, including stage-specific survival rates, especially on a population basis, are not. The inadequate system of registration of death and incomplete or incorrect certification of cause of death accompanied by poor patient follow-up are the main reasons for the paucity of valid mortality data and, therefore, lack of survival information on cancer patients. This has been largely overcome in this study.

### Materials and methods

Under the National Cancer Registry Programme of the Indian Council of Medical Research a population-based cancer registry (PBCR) was started from 1 January 1982 at Kidwai Memorial Institute of Oncology, Bangalore. The registry covers the area of Bangalore Urban Agglomeration (which includes Bangalore city) with a total population of 4.1 million (Census of India, 1991) and a male-female ratio of 1:0.9. The criterion for including cancer patients in the registry is that the person should have resided in the registry area for a minimum period of 1 year before the diagnosis of cancer.

Kidwai Memorial Institute of Oncology (KIMIO) is a comprehensive cancer centre and a referral hospital for cancer patients with all modern facilities for diagnosis and treatment. Consequently, it is the most important single source of registration of cancer cases in the PBCR area and accounted for 65.9% of cases of cancers of all sites and 87.3% of cancer cervix during the above period. Therefore, this proportion of patients from the registry area get automatically registered with the registry and all details pertaining to diagnosis and treatment become readily available. Trained registry staff make periodic visits to other hospitals, nursing homes and histopathology laboratories (numbering in all about 30 major institutions and 200 smaller ones) in the registry area of Bangalore Urban Agglomeration to abstract details of cancer cases. Information so obtained in a core proforma (the format of which is computerised) is entered on a personal computer and duplicate and consistency checks are carried out. The abstraction of the extent of disease and stage is always carried out by a medical officer who is familiar with clinical staging in oncology.

Records of deaths from all causes are scrutinised at 14 municipal corporation units, and information on cancer deaths is abstracted. By means of personal data (name, age, sex, etc.) these records are matched with the incident cases obtained through hospitals and other sources. The matched records constitute the matched deaths. The unmatched ones are those considered as 'death certificates only' (DCO) and by convention are added to the incident cases of the relevant year in calculating incidence rates, but are normally excluded from survival analysis, since only the date of death of these cases would be available and consequently their survival time would be zero.

Taking into account the proportion (74.1% in males and 58.8% in females) of literates in the population (Nandakumar *et al.*, 1990), their health consciousness, the health facilities available in the area and the cooperation extended by these institutions, it is estimated that the coverage by the registry would be over 90%.

Thus all patients (2423 cases) with cervical cancer (ICD: 180) diagnosed between 1 January 1982 and 31 December 1989 and registered in the PBCR of Bangalore constituted the study group. Information that was available with the registry included: date of diagnosis, area of residence and duration of stay, age at diagnosis, most valid basis of diagnosis, educational and marital status, religious group and clinical extent of disease before treatment. In order to obtain a better assessment of the clinical extent at presentation, the case records of all patients with cancer of the cervix were once again reviewed for abstracting information on pre-

Correspondence: A Nandakumar
Received 19 September 1994; revised 10 January 1995; accepted 27 January 1995

treatment composite FIGO (International Federation of Gynaecology and Obstetrics) stage at presentation. This was available in 88.9% of patients. Since over 90% of patients in each of the stages were classified as subgroup B at presentation, no attempt was made to subclassify further each of the four stages under the FIGO classification into subgroups A and B.

Age-specific rates and ASRs including trends over time were calculated for all 2423 incidence cases that were registered. Age-specific and age-standardised mortality rates including trends over time were calculated for 1076 deaths that occurred between 1 January 1982 and 31 December 1989. Standardisation of incidence and mortality rates was achieved by the direct method using the World Standard Population (Boyle and Parkin, 1991). The age at diagnosis and age at death were taken for calculating age-specific and age-standardised incidence and mortality rates respectively. The population of Bangalore Urban Agglomeration by 5 year age group and sex was estimated (as on 1 July of each intercensal year) using the 1971 and 1981 quinquennial population by sex and the total population of 1991 also by sex (Census of India, 1971, 1981).

The estimated proportion of different religious groups for the total population were available (Hindus, 76.5%; Muslims, 15.2%; Christians, 7.2%; and others, 1.1%), therefore crude incidence rates according to religion could be calculated. However, the proportions of 5 year age distribution of the populations by religious groups were not available and therefore the age-standardised rates according to religious groups could not be calculated.

Active follow-up through visits to the homes of patients was performed by trained social investigators. The detailed methodology of active follow-up is described elsewhere (Nandakumar, 1993). Briefly, matched deaths, which constituted 7.6% (185 cases) of the total cases and 2.3% (55 cases) of patients who were on attendance at KIMIO, were excluded from active follow-up. Information from home visits and the last hospital attended was obtained for the 45 cases for whom death certificates were the only records that were available as these did not match the records of other cases of cervical cancer that were registered. House visits were attempted for the remaining 2138 cases. January 1 1993, was taken as the cut-off date for survival analysis, and the vital status (whether alive or dead) on that date was determined through these visits. Patients who had died during 1993 but were alive on 1 January 1993 were considered alive for the purpose of this analysis.

Active follow-up of 2138 cases yielded information on vital status in 1704 cases, partial follow-up information in 161 cases and no follow-up information in the remaining 273 cases. Of the 45 unmatched death certificate only (DCO) cases that were 'followed back', the date of first diagnosis was obtained in 16, and these were considered as matched deaths, whereas the remaining 29 cases were considered as DCO cases.

Thus, these 29 DCO cases, along with 273 cases for whom no follow-up information subsequent to the date of diagnosis was available, were excluded from survival analysis, leaving 2121 of 2423 (87.5%) cases for calculating survival. For the 161 patients in whom only partial follow-up was available it was assumed that these patients were alive for exactly half the period since they were last traced (Parkin and Hakulinen, 1991).

Observed survival was based on death from all causes. The overall observed survival and that according to 10 year age group, year of diagnosis, clinical stage (FIGO system of staging), religious group and literary status were computed using the Kaplan–Meier method of calculating survival (Kaplan and Meier, 1958). The effects of age and mortality from all causes of death (Corporation of the City of Bangalore, 1986, 1987) were removed by computing survival relative to that expected in the total population of Bangalore by age and sex using the life table method (Shryock and Siegel, 1973). The factored variables that showed statistical significance were introduced stepwise into a Cox regression model (Cox, 1972) to remove the effects of one against the other. Both survival and Cox's proportional hazard ratio were calculated using the EGRET software package. Since never married cases constituted less than 0.5% of the cases, survival by marital status was not calculated.

### Results

*Incidence and mortality*

The average annual crude and age-standardised incidence rate (ASR) of cancer of the cervix in Bangalore for the period 1982–89 was 18.6 and 28.8 per 100 000 respectively. Over the 8 year period there appeared to be a decrease in the ASR, and this was statistically significant ($P<0.02$). The average annual crude and age-adjusted death rate for the period were 8.2 and 13.3 per 100 000 respectively, giving a mortality incidence ratio of 0.46.

The age-specific incidence and mortality rates are diagrammatically shown in Figure 1. While the highest age-specific incidence rate of 106.2 per 100 000 was seen in the 55–59 year age group, the highest age-specific mortality rate of 54.1 per 100 000 was seen in the 65–69 year age group.

Crude incidence (CR) and mortality rates according to religious groups were examined. The CR among Hindus (21.2 per 100 000) was almost three times higher than in Muslims (CR = 7.7 per 100 000). The corresponding CR among Christians was 15.9 per 100 000. There was no variation in the mortality incidence ratio between Hindus and Muslims (0.44). In Christians this ratio was 0.51. Since age distribution of the population according to religious groups was not available, the age-adjusted rates could not be calculated.

*Incidence and mortality according to clinical stage* Table I gives the age-adjusted incidence and mortality rates according to clinical stage as well as the mortality incidence ratio. This ratio shows a rise with advancing stage of disease. The table also gives the number and proportions of patients with different stages of disease. If those patients with stage unknown were excluded, only 3.4% of patients presented with stage I disease and 68.6% of patients had stage III or IV disease. From 1982 to 1989 a trend in the relative proportion of each stage of disease was observed (Figure 2). During the 8 year period there was a statistically significant decline in

**Figure 1** Age-specific incidence and mortality rates.

the relative proportion of stage IV disease ($P = 0.01$) but no significant change in the relative proportion of other stages. Similarly, the trends in ASR according to clinical stage of disease showed a statistically significant decline in stage III and stage IV disease.

*Survival analysis*

The overall observed 5 year survival was 34.4% (Figure 3), and when the 5 year probability of survival (0.898) in the general population (among females of Bangalore) based on death from all causes was taken into account, the relative survival was 38.8%. Table II gives the patient characteristics and observed 5 year survival proportion. The influence of age on survival was observed by calculating the hazard ratio for both 5 and 10 year age groups. This was not statistically significant. Five year observed survival was almost identical in the different religious groups. The differences in survival seen between illiterate and literate patients on univariate analysis was not statistically significant when the clinical stage of disease was also considered during multivariate analysis (Table III). There was little difference in the observed survival of patients during different years of diagnosis.

Tables II and III show that clinical stage was a strong independent predictor of survival. The corresponding survival curves are shown in Figure 4.

**Figure 2** Trends over time: relative proportion according to clinical stage.

**Figure 3** Overall observed survival. Total cases = 2121.

### Discussion

India has one of the highest incidence rates of cancer of the cervix, and the ASR of this cancer in Bangalore is second only to that in Madras (Annual Reports, NCRP, ICMR; Parkin *et al.*, 1992). However, as observed in this study, there appears to have been a statistically significant decline in the incidence rate of cervical cancer in Bangalore. A decline in cervical cancer incidence has been reported in the western world (Miller *et al.*, 1992). One possible explanation for the

**Table I** Average age-adjusted incidence (IR) and mortality rates (MR) per 100 000, mortality incidence ratio (MIR) and number (No.) and proportion (%) according to clinical stage (FIGO classification) of cancer of the cervix

| *FIGO stage* | *IR* | *MR* | *MIR* | *No.* | *%* |
|---|---|---|---|---|---|
| Stage I | 0.85 | 0.25 | 0.29 | 74 | 3.1 |
| Stage II | 6.87 | 2.54 | 0.37 | 602 | 24.8 |
| Stage III | 16.62 | 8.04 | 0.48 | 1375 | 56.7 |
| Stage IV | 1.29 | 0.99 | 0.77 | 102 | 4.2 |
| Stage unknown | 3.15 | 1.45 | 0.46 | 270 | 11.1 |
| All stages | 28.79 | 13.27 | 0.46 | 2423 | 100.0 |

**Table II** Patient characteristics and observed 5 year survival

| *Patient characteristics* | *No. of patients* | *Five year survival (%)* |
|---|---|---|
| *Age group (years)* | | |
| <25 | 13 | – |
| 25–34 | 147 | 40.9 |
| 35–44 | 487 | 42.0 |
| 45–54 | 676 | 35.6 |
| 55–64 | 505 | 30.0 |
| 65–74 | 231 | 24.0 |
| 75+ | 62 | 16.0 |
| *Religious group* | | |
| Hindu | 1841 | 34.4 |
| Muslim | 134 | 34.0 |
| Christian | 143 | 34.5 |
| Others | 3 | – |
| *Educational status* | | |
| Illiterate | 1361 | 33.8 |
| Literate | 675 | 37.2 |
| *Clinical stage* | | |
| Stage I | 69 | 63.3 |
| Stage II | 556 | 44.0 |
| Stage III | 1227 | 30.3 |
| Stage IV | 88 | 5.7 |
| Unstaged | 181 | 37.2 |

**Table III** Independent predictors of observed survival using Cox proportional hazards regression analysis

| *Factor* | *Hazard ratio* | *95% CI* | *LRS* | *P-value* |
|---|---|---|---|---|
| *Religious group* | | | 2.3 | 0.52 |
| Hindu | 1.0 | – | | |
| Muslin | 0.9 | 0.8–1.1 | | |
| Christian | 1.0 | 0.8–1.2 | | |
| Others | 0.3 | – | | |
| *Educational status* | | | 4.1 | 0.043 |
| Illiterate | 1.0 | – | | |
| Literate | 0.9 | 0.8–1.0 | | |
| *Clinical extent* | | | 122.7 | <0.001 |
| Stage I | 1.0 | – | | |
| Stage II | 1.9 | 1.3–2.7 | | |
| Stage III | 2.6 | 1.8–3.8 | | |
| Stage IV | 6.4 | 4.3–9.7 | | |
| Unstaged | 2.3 | 1.6–3.4 | | |

CI, confidence interval; LRS, likelihood ratio statistic.

**Figure 4** Observed survival according to clinical stage.

decline of cancer of the cervix in developing countries as in Bangalore, is the increase in age at marriage amidst a changing socioeconomic environment. A decline in the incidence of cancer of the cervix has also been reported from Bombay (Jayant, 1986).

Examination of the decline in cervical cancer according to clinical stage was of interest. Thus the ASR of stage I and stage II disease showed no change during the 8 year period. However, the ASR of both stage III and stage IV disease declined. Similarly, when the trends in stage proportions for each calendar year were observed a statistically significant decline in proportion of stage IV disease was seen. Whether this is an indication of patients in the area seeking early diagnosis and treatment because of greater awareness of their disease and its symptoms remains to be determined.

Nearly 70% of patients presented with stage III disease or higher at diagnosis. Even in a developing country, such as here in India, there is a difference of 33% in observed 5 year survival between patients surviving stage I (5 year survival 63.3%) and stage III (5 year survival 30.3%) disease. Thus, there should be a corresponding improvement in 5 year survival if patients with stage III disease were to be diagnosed at stage I. This then is the concept of 'downstaging' for cancer of the cervix, in the context of the developing world (Stjernsward *et al.*, 1982), wherein a higher proportion of patients are diagnosed at an earlier stage of the disease, thereby giving an opportunity for curative treatments and consequent improved survival. A combination of professional and public education with improved diagnostic and therapeutic facilities would make possible this 'shift' to earlier stage at diagnosis.

The mortality rate presented for all the years combined is an underestimate of the true picture of mortality due to cervical cancer in the population. This is mainly because the method used to collect information on vital status did not allow details of deaths to be obtained for patients who were diagnosed as having cervical cancer before commencement of the registry in 1982, and who died during 1982 or later. Thus, the average (18.0/100 000) age-standardised mortality rates for the last three years (1987, 1988, 1989) of this study are a better reflection of the mortality due to this disease than the average age-standardised mortality rate (13.3/100 000) of the entire 8 year period. The mortality/incidence ratio calculated for the average of these last three years is 0.66, which is fairly high compared with that seen in the western world. Miller *et al.* (1992) and Giles *et al.* (1993) in their data from the US surveillance epidemiology end results (SEER) and Victorian Cancer Registry report these ratios as 0.33 and 0.31 respectively.

The inadequate system of registration and certification of cause of death is a barrier to determining mortality. In addition, inadequate follow-up of patients makes it very difficult to obtain information on survival. In the West the term 'follow-up' refers to the reassessment of disease status and, if necessary, further treatment by the treating doctor of patients who have completed initial or subsequent cancer-directed treatment. However, in India the term 'follow-up' is also applied to patients who are lost even before investigations are done or are completed though they have a clinical diagnosis of cancer. It is also applied to patients in whom a diagnosis of cancer is established but who fail to turn up for treatment or are lost during treatment (the category of patients who do not comply with treatment) and to the group of patients who complete the initial and/or subsequent treatment but do not report to the treatment centre for a 'check-up'. Finally, no information is available on patients who have terminal or advanced cancer and who go home or are sent home to 'die' (Nandakumar *et al.*, 1990). The reasons for the above state of affairs are several and include lack of awareness about disease, economic factors and the poor quality of patient care, but then this is not the focus of this discussion. Suffice to say that the above serves to highlight the need to obtain adequate information on vital status through active follow-up in order to determine reliable survival estimates.

The results on survival for a developing country such as India are not unexpected. The 5 year relative survival of 38.3% is low compared with that seen in Scotland (58.7%) (Black *et al.*, 1993) and that in the United States (68% in whites and 60% in blacks) (Miller *et al.*, 1992). The proportion of patients presenting at a late stage of the disease is one reason for this comparatively low survival in Bangalore. The data presented here show that only 3.4% of women presented at localised or stage I disease compared with 48% in the SEER data of USA. However, when the survival of patients with stage I or localised disease is considered, 5 year survival in Bangalore is just 63.3% compared with 89.2% survival in the United States (Miller *et al.*, 1992). This suggests that aspects of treatment management including that of patients not complying with treatment, as mentioned above, have a bearing on outcome.

In our study age did not appear to influence relative survival, nor did the survival change because of educational status or patient professing a particular religion. The difference in survival seen between literates and illiterates on univariate analysis but not after adjustment for stage in multivariate analysis suggests that education is probably only a confounder strongly associated with stage of disease. Improved survival was not observed during the 8 year period, reflecting the absence of primary and secondary prevention programmes and also the lack of any impact, as yet, on patient care of the opening of several cancer therapy centres in the region. However, the lack of any apparent improvement in survival could reflect the relatively short study period.

A low incidence of cancer of the cervix has been reported in countries where the population is predominantly Muslim (Tomatis *et al.*, 1990), and this was also reflected in the registry data at Bangalore, where the incidence of cervical cancer among Muslims was less than half that seen in Hindu females. However, the fact that mortality or survival was not different amongst the religious groups suggests that one is probably dealing with the same biological disease.

The incidence, mortality and survival figures on a population basis provide baseline data for cancer control programmes of this common, important preventable cancer. The added component of clinical stage of disease would help in evaluating screening programmes, whether they be screening through cytology or only clinical examination. In the absence of any such data elsewhere in the country or region the figures of various rates and proportions presented in this paper provide a starting point for designing, implementing and, to a certain extent, evaluating prevention and treatment programmes in other parts of the state and country.

**Acknowledgement**
The authors gratefully acknowledge the assistance provided by the Descriptive Epidemiology Unit (Chief: Dr DM Parkin) of the International Agency for Research on Cancer, Lyon, France, where Dr Nandakumar spent two weeks on a survival analysis of breast cancer.

**References**

ANNUAL REPORTS OF THE NATIONAL CANCER REGISTRY PROGRAMME (NCRP) OF INDIA. (1982-1990). Indian Council of Medical Research: New Delhi.

BLACK RJ, SHARP L AND KENDRICK SW (eds). (1993). *Trends in Cancer Survival in Scotland, 1968-1990*. Information and Statistics Division, Directorate of Information Services, National Health Service in Scotland: Edinburgh.

BOYLE P AND PARKIN DM. (1991). Statistical methods for registries. In *Cancer Registration: Principles and Methods*, IARC Scientific Publications No. 95, Jensen OM, Parkin DM, Maclennan R, Muir CS and Skeet RG. (eds). pp. 126-158. IARC: Lyon.

CENSUS OF INDIA PUBLICATION. (1971, 1981, 1991). Karnataka, Director of Census, Bangalore, India.

CORPORATION OF THE CITY OF BANGALORE. (1986, 1987). *Report*. Bangalore, India.

COX DR. (1972). Regression models and life tables. *J.R. Stat. Soc. B*, **34**, 187-220.

GILES G, FARRUGIA H, THURSFIELD V AND STAPLES M. (1993). *Canstat*, Publication No. 17 of Cancer Epidemiology Centre. Anti-Cancer Council of Victoria: Carlton South, Australia.

JAYANT K. (1986). Cancers of the cervix, uteri and breast: changes in incidence rates in Bombay over the last two decades. *Bull. WHO*, **64**, 431-435.

KAPLAN EL AND MEIER P. (1958). Non parametric estimation from incomplete observations. *J. Am. Stat. Assoc.*, **53**, 457-481.

MILLER AB, GLOECKLER RIES LA, HANKEY BF, KOSARY CL AND EDWARDS BK. (1992). *Cancer Statistics Review, 1973-1989*, NIH Publication No. 92-2789, pp. 1-3, 12-17. US Department of Health and Human Services: Bethesda, MD.

NANDAKUMAR A. (1993). Strategy for active patient follow-up in the conduct of survival studies, presented at Annual Review Meeting of National Cancer Registry Programme of India (ICMR), Dibrugarh India, 24-26 November 1993.

NANDAKUMAR A, ANANTHA N AND THIMMASETTY K. (1990). *Cancer Patterns in Bangalore, India 1982-89*. Population Based Cancer Registry of Bangalore, Kidwai Memorial Institute of Oncology: Bangalore, India.

PARKIN DM AND HAKULINEN T. (1991). Analysis of survival. In *Cancer Registration: Principles and Methods*, IARC Scientific Publications No 95, Jensen OM, Parkin DM, Maclennan R, Muir CS and Skeet RG. (eds). pp. 159-176. IARC: Lyon.

PARKIN DM, MUIR CS, WHELAN SL, GAO YT, FERLAY J AND POWELL J. (1992). *Cancer Incidence in Five Continents*, Vol VI, IARC Scientific Publications No 120. IARC: Lyon.

SHRYOCK HS AND SIEGEL JS. (1973). *The Methods and Materials of Demography*. US Bureau of Census, US Printing Office: Washington DC.

STJERNWARD J, EDDY D, LUTHRA UK AND STANLEY K. (1982). Plotting a new course for cervical cancer screening in developing countries. *World Health Forum.*, **8**, 42.

TOMATIS L, AITIO A, DAY NE, HESELTINE E, KALDOR J, MILLER AB, PARKIN DM AND RIBOLI E. (1990). *Cancer: Causes, Occurrence and Control*, IARC Scientific Publications No 100. IARC: Lyon.

WHELAN SL, PARKIN DM AND MASUYER E. (1990). *Patterns of Cancer in Five Continents*, IARC Scientific Publications No 102. IARC: Lyon.